# Comparison of salivary gland and midgut microbiome in the soft ticks *Ornithodoros erraticus* and *Ornithodoros moubata*

**DOI:** 10.3389/fmicb.2023.1173609

**Published:** 2023-05-09

**Authors:** Elianne Piloto-Sardiñas, Ana Laura Cano-Argüelles, Apolline Maitre, Alejandra Wu-Chuang, Lourdes Mateos-Hernández, Alexandra Corduneanu, Dasiel Obregón, Ana Oleaga, Ricardo Pérez-Sánchez, Alejandro Cabezas-Cruz

**Affiliations:** ^1^Direction of Animal Health, National Center for Animal and Plant Health, Carretera de Tapaste y Autopista Nacional, San José de las Lajas, Mayabeque, Cuba; ^2^ANSES, INRAE, Ecole Nationale Vétérinaire d’Alfort, UMR BIPAR, Laboratoire de Santé Animale, Maisons-Alfort, France; ^3^Parasitology Laboratory, Institute of Natural Resources and Agrobiology (IRNASA, CSIC), Salamanca, Spain; ^4^UR 0045 Laboratoire de Recherches Sur Le Développement de L’Elevage (SELMET-LRDE), INRAE, Corte, France; ^5^EA 7310, Laboratoire de Virologie, Université de Corse, Corte, France; ^6^Department of Animal Breeding and Animal Production, University of Agricultural Sciences and Veterinary Medicine, Cluj-Napoca, Romania; ^7^Department of Parasitology and Parasitic Diseases, University of Agricultural Sciences and Veterinary Medicine, Cluj-Napoca, Romania; ^8^School of Environmental Sciences University of Guelph, Guelph, ON, Canada

**Keywords:** microbiome, salivary gland, midgut, *Ornithodoros erraticus*, *Ornithodoros moubata*, networks

## Abstract

**Introduction:**

*Ornithodoros erraticus* and *Ornithodoros moubata* are the main vectors of African swine fever virus (ASFV) and the human relapsing fever spirochetes *Borrelia hispanica* and *Borrelia crocidurae* in the Mediterranean region and *Borrelia duttoni* in continental Africa. Manipulation of the tick microbiome has been shown to reduce vector fitness and competence in tick vectors, suggesting that the identification of key microbial players associated with tick tissues can inform interventions such as anti-microbiota vaccines to block pathogen development in the midgut and/or salivary glands.

**Methods:**

In this study, we analyzed and compared the microbiome of the salivary glands and midgut of *O. erraticus* and *O. moubata*. For the taxonomic and functional characterization of the tissue-specific microbiome, we used 16S rRNA amplicon sequencing and prediction of metabolic profiles using PICRUSt2. Co-occurrence networks were built to characterize the community assembly and identify keystone taxa in each tick species.

**Results:**

Our results revealed differences in the composition, diversity, and assembly of the bacterial microbiome of salivary glands and midgut within each tick species, but differences were more noticeable in *O. moubata*. Differences were also found in the microbiome of each tissue, salivary gland and midgut, between species. However, the ‘Core Association Networks (CAN)’ analysis revealed conserved patterns of interacting taxa in tissues within and between tick species. Different keystone taxa were identified in *O. erraticus* and *O. moubata* tissues, but Muribaculaceae and *Alistipes* were found as keystone taxa in the salivary glands of both tick species which justifies their use as anti-microbiota vaccine candidates to alter the microbiome and reduce tick fitness and/or block pathogen transmission. The high similarity of predicted metabolic pathways profiles between tissues of the two tick species suggests that taxonomic variability of the microbiome is not associated with significant changes in microbial functional profiles.

**Conclusion:**

We conclude that the taxonomic structure of the microbiome in *O. erraticus* and *O. moubata* is tissue-specific, suggesting niche partitioning of bacterial communities associated to these soft ticks. However, shared keystone taxa and conserved patterns of interacting taxa between tissues and tick species suggest the presence of key microbial players that could be used as anti-microbiota vaccine candidates to affect tick physiology and/or pathogen colonization.

## 1. Introduction

Ticks are a growing medical and veterinary concern because tick feeding cause direct harm to their hosts, but more importantly because ticks are efficient vectors of numerous pathogens that cause severe diseases in wildlife, livestock, pets and humans, resulting in significant economic losses worldwide (Rashid et al., 2019). Ticks are haematophagous arthropods belonging to two major families: Ixodidae (hard ticks) and Argasidae (soft ticks). Ixodids are usually exophilic ticks that persist on the soil and in vegetation, actively searching for hosts when the season is suitable. After attaching to a suitable host, they feed for several days, ingest large quantities of blood, and, after becoming engorged, drop from the host and return to the soil, where they moult or, in the case of females, oviposit and die. In contrast, argasid ticks are endophilic/nidicolous parasites. In nature, they live in nests and burrows of their hosts, while in synanthropic environments they colonize animal facilities and human dwellings, hiding in the floor, walls, and ceiling. There they are protected from adverse climatic conditions and have regular access to host blood. Most argasids are fast feeders, taking less than an hour to complete engorgement. They then drop off the host and hide in their shelters to moult or reproduce. Adult specimens can feed and reproduce repeatedly and are very resistant to starvation and can survive for years without food (Sonenshine and Roe, 2014).

Among the Argasid ticks, several *Ornithodoros* species are vectors of microbial pathogens that cause severe diseases. *Ornithodoros erraticus* and *Ornithodoros moubata* are the main vectors of the African swine fever virus (ASFV) and the human relapsing fever spirochetes *Borrelia hispanica* and *Borrelia crocidurae* in the Mediterranean and *Borrelia duttoni* in mainland Africa (Arias et al., 2018; Talagrand-Reboul et al., 2018). The presence of *Ornithodoros* vectors in anthropogenic environments is a challenge for the eradication of ASFV and tick-borne relapsing fever (TBRF) in endemic areas, especially in Africa. These soft ticks also contribute to the spread of ASFV to new territories from Eastern Europe to China (Sánchez-Vizcaíno et al., 2021), as well as increase the risk of reintroduction of ASFV to countries such as Spain and Portugal where this virus has already been eradicated (Arias et al., 2018). Therefore, an effective strategy for the prevention and control of ASFV and TBRF should include the elimination of *Ornithodoros* vectors from at least the anthropogenic environment.

Most tick control strategies rely on the application of chemical acaricides, but this selects resistant tick strains and causes chemical residues to accumulate in animal products and the environment (Abbas et al., 2014). Alternative methods of tick control are urgently needed, and tick vaccines have proven to be an effective and sustainable method of controlling tick infestations and tick-borne diseases (Valle and Guerrero, 2018; Ndawula and Tabor, 2020).

Many tick vaccine development studies have focused on identifying and testing molecular components of tick physiological pathways as targets for vaccine candidates. The tick salivary glands (SG) and midgut (MG) are important components of the interface between host-tick-pathogen and play an important role in tick survival, reproduction and pathogen transmission. Accordingly, numerous tick antigens derived from these tissues have been tested as vaccine candidates (Van Oosterwijk and Wikel, 2021). Regarding *Ornithodoros* sp. vectors, previous studies has identified several salivary and intestinal antigens that could be incorporated into the development of multi-antigen anti-*Ornithodoros* vaccines, furthering the search for new and more effective antigen targets (Díaz-Martín et al., 2015; Obolo-Mvoulouga et al., 2018; Pérez-Sánchez et al., 2019a,b).

Currently, advances in defining the tick microbiome are providing new insights into tick physiology, reproduction, supply of essential molecules not synthesized by the tick itself, tick-host-pathogen interaction and vector competence (Aguilar-Díaz et al., 2021; Narasimhan et al., 2021). This increasing knowledge of microbial ecology and vector-host interactions is leading to new concepts and methods for vector and pathogen control, including anti-tick microbiota vaccines (Mateos-Hernández et al., 2021; Maitre et al., 2022; Wu-Chuang et al., 2022a). The rationale for anti-tick microbiota vaccination is that tick transmission and tick-borne pathogens can be controlled by disrupting bacterial taxa that are central to supporting other microbiome species and essential physiological functions of the tick (Maitre et al., 2022). The innovative study by Mateos-Hernández et al. (2020) showed that host antibodies induced by immunization with a tick microbiome Enterobacteriaceae caused significant mortality of engorging ticks, opening the way for the development of novel anti-tick and transmission-blocking vaccines.

Accordingly, the aim of the present study was to characterize and compare the bacterial microbiome of the SG and MG of *O. erraticus* (abbreviated here as OeSG and OeMG, respectively) and *O. moubata* (abbreviated here as OmSG and OmMG, respectively). Our results revealed that despite differences in the diversity and assembly of the tissue-associated bacterial microbiome of soft ticks there were conserved keystone taxa that could be used as anti-microbiota vaccine candidates to affect tick physiology and/or pathogen colonization.

## 2. Materials and methods

### 2.1. Ticks and tick material

The specimens of *O. moubata* (Om) and *O. erraticus* (Oe) used in this study were obtained from two laboratory colonies maintained at the IRNASA insectarium (CSIC). The *O. moubata* colony was initiated from specimens kindly donated by Dr. Philip Wilkinson (Institute for Animal Health, Pirbright, United Kingdom), originally from Malawi (13° 59′ 00″ S 33° 47′ 00″ E). The *O. erraticus* colony was formed from specimens captured in the wild in the province of Salamanca (Spain; 40° 58′ 00″ N 5° 39′ 50″ O). Both colonies were established in the IRNASA insectarium in the 1990’s. Since then, they have been routinely maintained at 28°C and 85% relative humidity and are regularly fed on rabbits.

The procedures for tick feeding and the experiments with rabbits were approved by the Ethical and Animal Welfare Committee of the Institute of Natural Resources and Agrobiology (IRNASA) and the Ethical Committee of the Spanish National Research Council (CSIC, Spain; Permit Number 742/2017) and complied with the relevant EU legislation (Directive 2010/63/EU).

### 2.2. DNA extraction from midgut and salivary glands

Prior to dissection and deoxyribonucleic acid (DNA) extraction, the ticks were washed once with ethanol 70% and once with hydrogen peroxide 3%, to disinfect their surface. Unfed female ticks of *O. moubata* (*n* = 100) and *O. erraticus* (*n* = 100) were dissected in ice-cold PBS, pH 7.4, and their salivary glands (SG) and midgut (MG) were carefully removed for genomic DNA extraction using the NucleoSpin® Tissue Kit (Macherey-Nagel, Germany). Ten samples of 10 pairs of SG or 10 MG *per* sample from each species were resuspended separately in T1 buffer and Proteinase K, then mechanically homogenized and incubated overnight at 56°C. Genomic DNA was then extracted according to the kit instructions. DNA concentration and quality were determined by spectrophotometry using the NanoDrop 2000c (Thermo Scientific, USA) and agarose gel electrophoresis.

### 2.3. 16S Rrna amplicon sequencing and processing of raw sequences

Genomic DNA was extracted and purified as previously described. A single lane of the Illumina MiSeq system was used to generate 251-base paired-end reads from variable region V4 of the 16S rRNA gene using barcoded universal primers (515F/806R) in ticks. The paired 16S rRNA raw sequences obtained from the OeMG, OeSG, OmMG, and OmSG samples were deposited in the SRA repository (Bioproject No. PRJNA931807).

Analysis of 16S rRNA sequences was performed using the QIIME 2 pipeline (v. 2021.4; Bolyen et al., 2019). The raw sequences (demultiplexed in fatsq fles), were denoised, quality trimmed and merged using the DADA2 software (Callahan et al., 2016) implemented in QIIME2 (Bolyen et al., 2019). The obtain amplicon sequence variants (ASVs) were aligned with q2-alignment of MAFFT (Katoh et al., 2002) and used to generate a phylogeny with q2-phylogeny of FastTree 2 (Price et al., 2010). Taxonomy was assigned to ASVs using a classify-sklearn naïve Bayes taxonomic classifier based on SILVA database (release 138; Bokulich et al., 2018). Only the target sequence fragments were used for the classifier (i.e., the classifier was trained with primers 515F/806R; Werner et al., 2012; Ren and Wu, 2016).

### 2.4. Diversity and differential taxonomic composition

The composition and diversity of the tissue-associated microbiome were compared within and between tick species. Microbial diversity analyses were performed using rarefied ASV tables calculated with the q2-diversity plugin in QIIME 2 (Bolyen et al., 2019). Alpha diversity richness was explored using observed features (DeSantis et al., 2006) and Faith’s phylogenetic diversity index (Faith, 1992), while evenness was explored with the Pielou evenness index (Pielou, 1966). Differences in alpha diversity metrics between groups were assessed with the Kruskal–Walli’s test (*p* ≤ 0.05) using QIIME 2 (Bolyen et al., 2019). Bacterial beta diversity was assessed with the Bray–Curtis dissimilarity index (Bray and Curtis, 1957) and compared between groups with the PERMANOVA test (*p* ≤ 0.05) on QIIME 2. Beta dispersion was calculated using the betadisper function and the Vegan script implemented in RStudio (Oksanen et al., 2021). Statistical analyses of beta dispersion were performed using an ANOVA test (*p* ≤ 0.05). Cluster analysis was performed with the Jaccard coefficient of similarity using Vegan (Oksanen et al., 2021) implemented in RStudio (RStudio Team, 2020). Jaccard distance is represented between 0 and 2, and lines are proportional to this distance.

Differences in taxa abundance between the four conditions were tested using a Kruskal Wallis test and implemented using the ANOVA-like differential expression (ALDEx2) package (Fernandes et al., 2013) on RStudio (RStudio Team, 2020). The ALDEx2 method involves constructing Monte Carlo samples of Dirichlet distributions to account for the uncertainty in the number of reads in each sample. Proportional data were then transformed using the centered log ratio (clr) transformation, so that standard statistical methods could be used. The identified differentially abundant taxa were used to create a heatmap in the RStudio (RStudio Team, 2020).

### 2.5. Inference of bacterial co-occurrence networks

Co-occurrence networks were created for each dataset using the taxonomic profiles at genera level. The networks provide a graphical representation of the microbial communities, with nodes representing taxa and edges indicating significant correlations between them. Analyses of significant positive (weight > 0.75) or negative (weight < −0.75) correlations were performed using the Sparse Correlations for Compositional data (SparCC) method (Friedman and Alm, 2012), implemented in RStudio (RStudio Team, 2020). Visualization and measurement of topological features (i.e., number of nodes and edges, network diameter, modularity, average degree, weighted degree and clustering coefficient) of the networks were performed using Gephi v0.9.2 (Bastian et al., 2009). Four Core Association Networks (CAN) were computed to search for conserved interactions in networks of different species and same tissues (i.e., OeMG vs. OmMG, and OeSG vs. OmSG) and networks of the same species and different tissues (i.e., OeMG vs. OeSG, and OmMG vs. OmSG) using the software toolbox, anuran (a toolbox with null models for identification of nonrandom patterns in association networks; Röttjers et al., 2021), and in a version available for Python 3.6.

#### 2.5.1. Differential network analysis

To compare OmMG, OmSG, OeSG, and OeMG networks, a statistical network estimation analysis was performed using the network construction and comparison for microbiome (NetCoMi) method (Peschel et al., 2021) implemented in RStudio (RStudio Team, 2020). To test for dissimilarities between the two networks (i.e., OmMG vs. OmSG), the Jaccard index was calculated to test for dissimilarities between nodes in the two networks for degree, betweenness centrality, closeness centrality and eigenvector centrality. The Jaccard index tests for the similarity between sets of “most central nodes” of networks, which are defined as those nodes with a centrality value above the empirical 75% quartile. This index expresses the similarity of the sets of most central nodes as well as the sets of hub taxa between the two networks. The Jaccard index ranges from 0 (completely different sets) to 1 (sets equal). The two *value of p*s P (J ≤ j) and P (J ≥ j) for each Jaccard index are the probability that the observed value of Jaccard’s index is ‘less than or equal’ or ‘higher than or equal’, respectively, to the Jaccard value expected at random which is calculated taking into account the present total number of taxa in both sets (Real and Vargas, 1996). The ARI was calculated to test the dissimilarity of clustering in the networks. The ARI values range from −1 to 1. Negative and positive ARI values mean lower and higher than random clustering, respectively. An ARI value of 1 corresponds to identical clustering, and 0 to dissimilar clustering. The value of p tests whether the calculated value is significantly different from zero (Peschel et al., 2021).

#### 2.5.2. Keystone taxa identification

Keystone taxa were defined based on three criteria, as previously reported (Mateos-Hernández et al., 2021): (i) ubiquitousness (microbial taxa present in all samples in an experimental group), (ii) eigenvector centrality higher than 0.75, and (iii) high mean relative abundance (i.e., higher than that of the mean relative abundance of all taxa in an experimental group). The eigenvector centrality measures the influence of a node in a network, a high eigenvector score means that a node is connected to many nodes which themselves have high scores (Ruhnau, 2000). Eigenvector centrality values were exported from Gephi (v 0.9.2) software (Bastian et al., 2009). For each sample in both datasets, the mean clr value was calculated and plotted together with the eigenvector centrality values using GraphPad Prism (v 8.0.0; GraphPad Software, San Diego, California USA).

#### 2.5.3. Network robustness in nodes removal

In this analysis, the proportion of removed nodes required to achieve a connectivity loss of 0.80 was recorded for the OeMG, OeSG, OmMG, and OmSG networks after directed, cascading, degree or random node removal. Directed removal of nodes consists in removing first the nodes with higher betweenness centrality. The cascading effect is that the nodes with high betweenness centrality are removed first, but recalculated each time a node is removed. The robustness of the network was also measured after removing nodes with very high degree. The last type is a random removal of nodes. The robustness of the networks is calculated using the Network Strengths and Weaknesses Analysis (NetSwan) script (Lhomme, 2015) in RStudio (RStudio Team, 2020).

### 2.6. Prediction and comparison of functional traits in the tick microbiome

The 16S rRNA amplicon sequences from each dataset were used to predict the metabolic profile of each sample. PICRUSt2 (Douglas et al., 2020) implemented in QIIME2, was used to predict the metagenomes from the 16S rRNA amplicon sequences. The ASVs were inserted into a reference tree (NSTI cut-of value of 3) containing more than 20,000 complete 16S rRNA sequences from prokaryotic genomes and then used to predict the copy numbers of each gene family for each ASV. The predictions are based on Kyoto Encyclopedia of Genes and Genomes (KEGG) orthologs (KO; Kanehisa, 2000). Pathways were generated based on the Meta-Cyc database (Caspi et al., 2018).

The differentially frequent paths were identified based on log_2_ fold change (LFC), using the Wald test implemented in the DESeq2 (v1.38.2) R package (Love et al., 2014). The DESeq2 pipeline was developed for differential gene analysis on smaller RNA-seq datasets with high overdispersion. The method assumes that the count data has a negative binomial distribution and models it using the maximum likelihood estimation (MLE) method. It includes a dispersion shrinkage estimation and fold-change estimation for each feature, resulting in accurate false discovery rates and FDR-corrected value of ps (Love et al., 2014).

## 3. Results

### 3.1. Differential bacterial composition and abundance in salivary glands and midguts of *Ornithodoros erraticus* and *Ornithodoros moubata*

**Figure 1:**
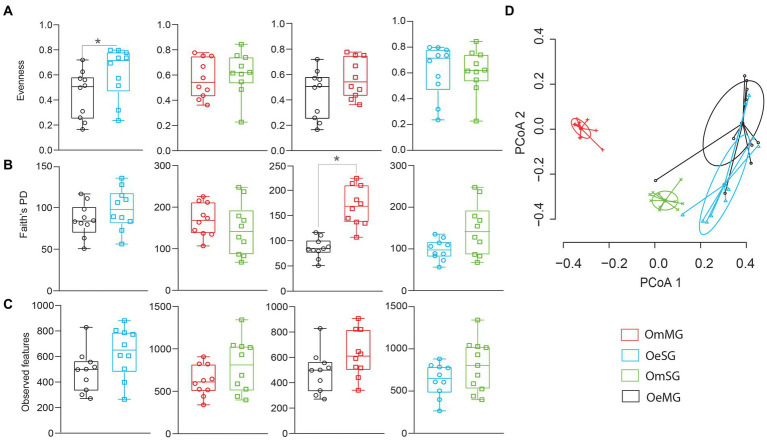
Comparison of microbial diversity for tissue-associated microbiome within and between *Ornithodoros erraticus* and *Ornithodoros moubata*. Comparison of alpha diversity for OeSG, OeMG, OmMG and OmSG samples, **(A)** Pielou evenness index, **(B)** Faith’s phylogenetic diversity (PD), **(C)** observed features. **(D)** Comparison of beta diversity with Bray–Curtis dissimilarity index for OeSG, OeMG, OmMG, and OmSG. Beta dispersion of four sets of samples (global comparison). Small circles, crosses and triangles represent samples, and ellipses represent dispersion of samples. This test use Principal Coordinate Analysis (PCoA), it is used to explore and to visualize variability in a microbial community. ANOVA test was performed and showed that beta dispersion of the two sets of samples (four conditions) is not significantly different (*p* = 0.849).

The alpha diversity of the salivary gland (SG) and midgut (MG) microbiome of *O. erraticus* (Oe) and *O. moubata* (Om) was compared using three diversity metrics (i.e., Pielou evenness index, Faith’s phylogenetic diversity index and observed features). Significant differences were found in the bacterial evenness of OeMG and OeSG (*p* < 0.05, Figure 1A), and in the phylogenetic diversity of OeMG and OmMG (*p* < 0.05, Figure 1B; Supplementary Table S1). No significant difference was found in the observed features within and between tissues of the two tick species (Figure 1C). Bray–Curtis index comparison showed that the composition of the tissue-associated microbiome was significantly different between tissues (SG and MG) in *O. moubata* and between species for the same tissue (SG or MG; PERMANOVA, *p* < 0.001, Figure 1D; Supplementary Table S1). However, Bray–Curtis index did not differ between tissues in *O. erraticus*. No significant differences were found in the beta dispersion of pairwise and global comparisons (ANOVA test, *p* > 0.05, Figure 1D), suggesting homogeneity of data dispersion among groups.

**Figure 2:**
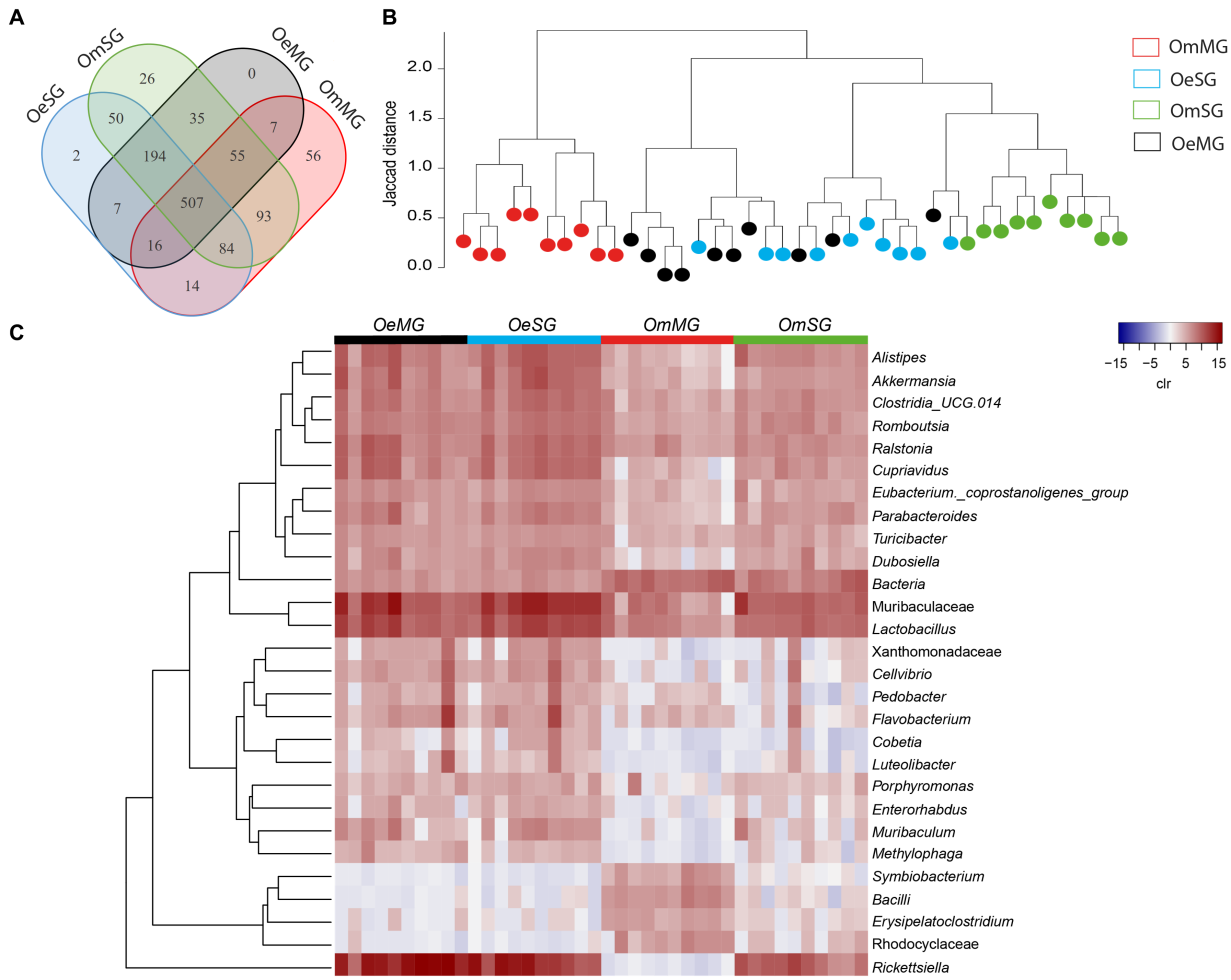
Comparison of microbial composition and abundance for the tissue-associated microbiome within and between *Ornithodoros erraticus* and *Ornithodoros moubata*. **(A)** Venn diagram displaying the comparison of taxa composition for all taxa. **(B)** Jaccard clusterization of OeMG, OeSG, OmMG and OmSG samples. The Jaccard distance is represented between 0 and 2, the lines are proportional to this distance. Each sample is represented by a color (legend). **(C)** Comparison of relative abundance for the tissue-associated microbiome within and between *O. erraticus* and *O. moubata*. Dendrogram heatmap resulting from the heatmap.2 function implemented on R studio. The taxa were clustered based on relative abundance (calculated as clr transformed values). Each column represents the clr values for bacterial taxa per sample and per group. Each line represents bacterial taxa with significant changes between the two datasets. Color represent the clr value.

Analysis of the bacterial composition in tissues of *O. erraticus* and *O. moubata* revealed a total of 1,146 bacterial taxa, of which 507 genera and families (44.2%) were shared by all samples (Figure 2A). In *O. moubata*, analysis revealed 82 unique bacterial taxa (7.16%), 56 in the MG (4.89%), and 26 in the SG (2.27%). Only two genera (0.17%) were identified as unique to the OeSG (Figure 2A; Supplementary Table S2). Hierarchical clustering of samples based on Jaccard distance showed that the microbiome of OmMG and OmSG clustered separately, while OeSG and OeMG clustered closely together (Figure 2B). Analysis of differential relative abundance (expressed as clr) showed that 30 taxa changed significantly between the four datasets (Figure 2C). Overall, these results suggest that soft tick microbiome is species-specific, and that differences in the bacterial composition and diversity between tissues is higher in *O. moubata* than in *O. erraticus*.

### 3.2. Assembly of microbial communities in the microbiome of salivary glands and midguts in *Ornithodoros erraticus* and *Ornithodoros moubata*

**Figure 3:**
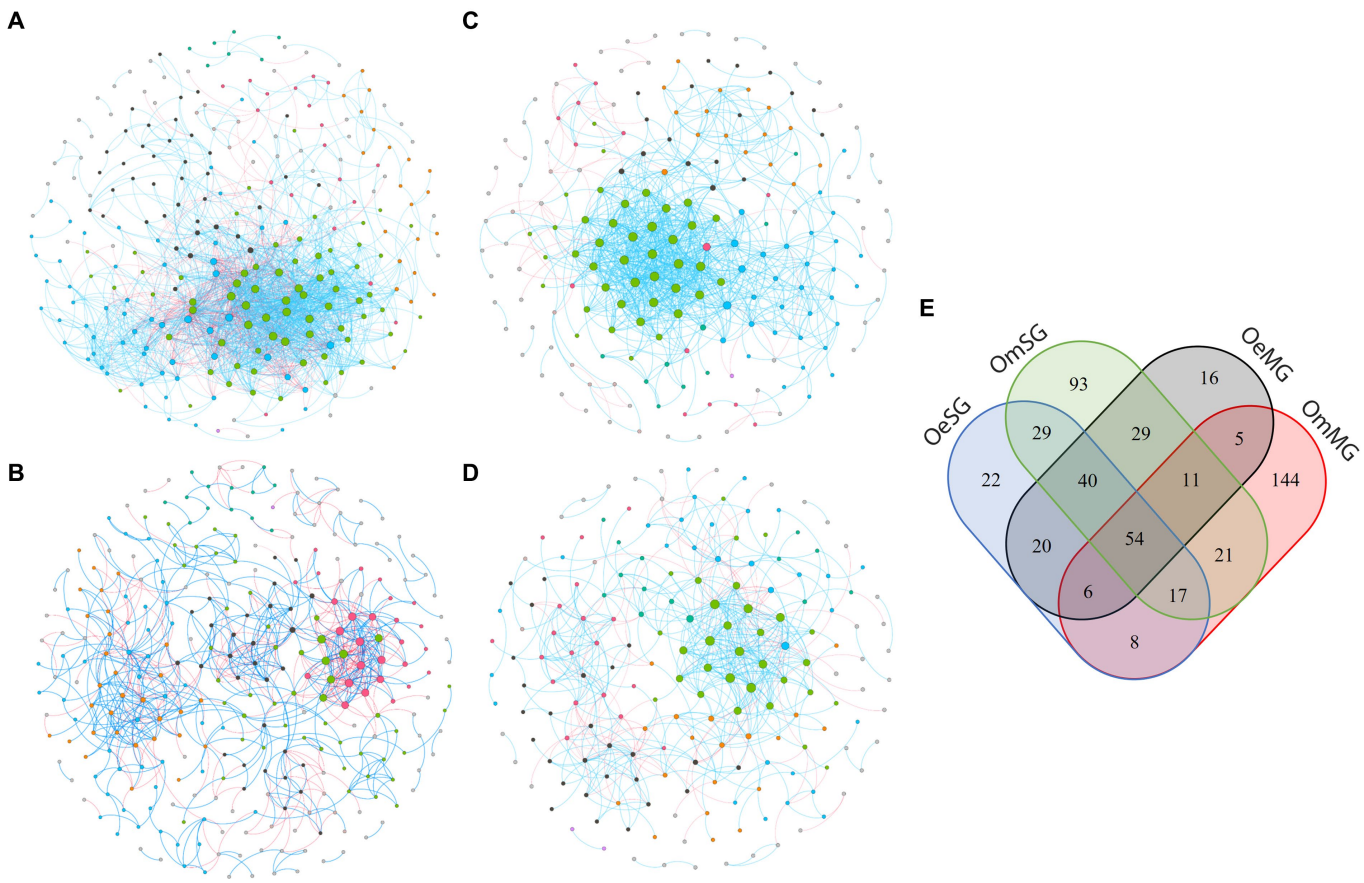
Co-occurrence networks of **(A)** OmMG, **(B)** OmSG **(C)** OeSG and **(D)** OeMG. Node colors are based on modularity class metric and equal color means modules of co-occurring taxa. The size of the nodes is proportional to the eigenvector centrality of each taxon. The colors in the edges represent strong positive (blue) or negative (red) correlations (SparCC >0.75 or <0.75). **(E)** Venn diagram displaying the comparison of networks composition.

Co-occurrence networks were constructed to characterize the assembly of the bacterial community in the four groups (Figure 3; Table 1). The networks showed that each tissue of the two tick species had different patterns of bacterial co-occurrence. The highest number of total and connected nodes was observed for OmMG (Figure 3A), while the highest number of edges, and the highest proportion of positive interactions were observed in OmSG (Figure 3B) and OeSG (Figure 3C) networks, respectively. The OeMG network (Figure 3D) has the lowest number of total nodes and connected nodes, positive interactions and edges. The average values for degree and clustering coefficient were higher for the OmMG network. The modularity value of the OmSG network was twice that of the OeSG network, while the diameter values were similar in all networks (Table 1). The highest number of unique nodes was found in OmMG, followed by OmSG, OeSG and OeMG, while 54 nodes were shared by all networks (Figure 3E; Supplementary Table S3).

**Table 1:** Topological features of the networks.

Topological features	OeMG	OeSG	OmMG	OmSG
Total nodes	821	874	832	1,001
Connected nodes	181	196	266	294
Edges	584	940	1783	793
Positives	449 (76.9%)	889 (94.6%)	1,312 (73.6%)	505 (63.7%)
Negatives	135 (23.1%)	51 (5.4%)	471 (26.4%)	288 (36.3%)
Modularity	0.93	0.41	0.87	2.02
Network diameter	12	11	11	15
Average degree	6.45	9.6	13.41	5.4
Weighted degree	2.86	7.1	5.18	1.2
Clustering coefficient	0.49	0.53	0.57	0.49

**Figure 4:**
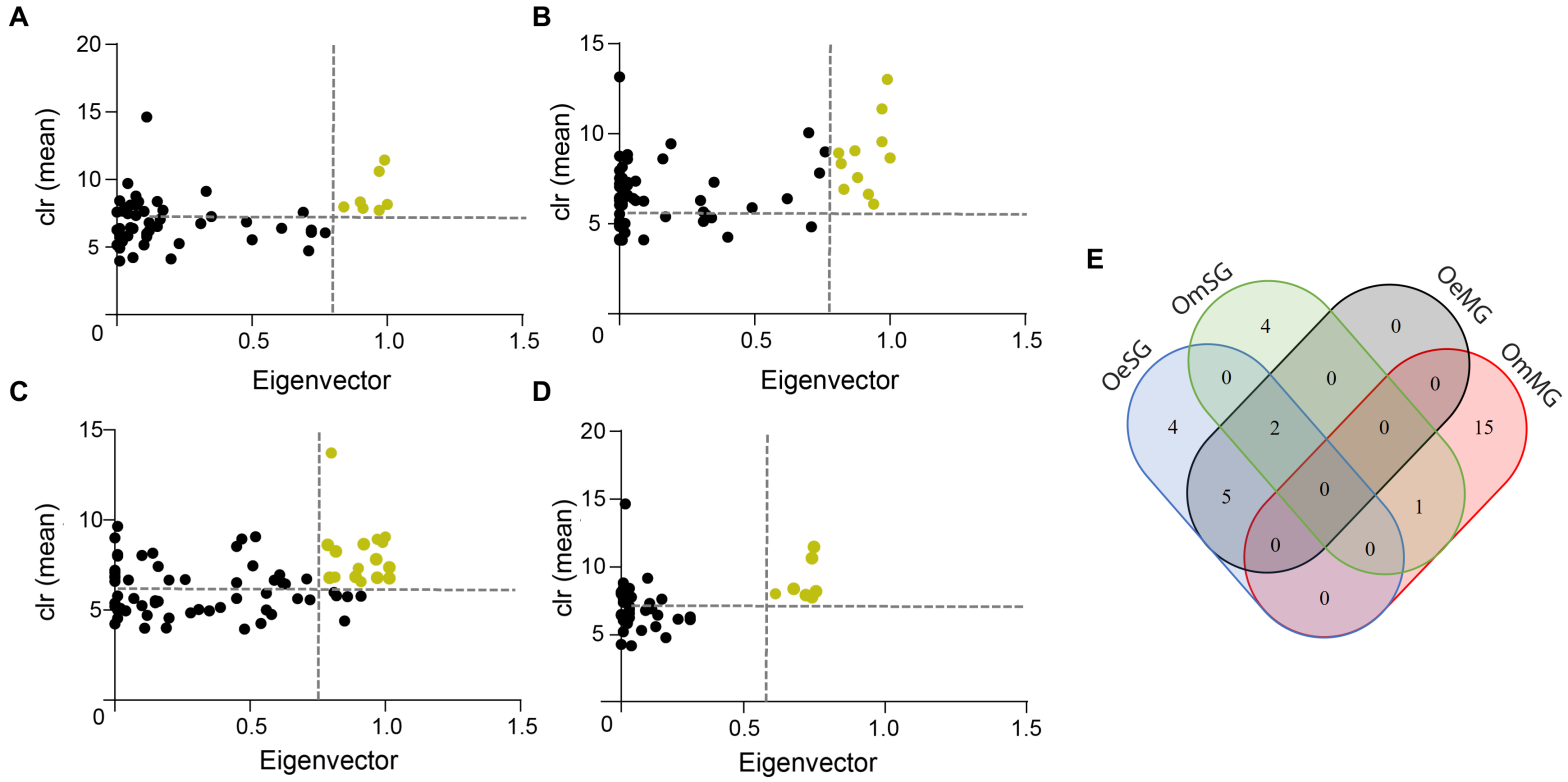
Keystone taxa in microbial communities. **(A)** OeMG, **(B)** OeSG, **(C)** OmMG and **(D)** OmSG datasets, showing the mean relative abundance (calculated as clr transformed values), and the eigenvector centrality of ubiquitous (present in all samples) bacterial taxa. The vertical dotted line represents the eigenvector centrality value cut-off set at 0.75 and the horizontal dotted line represents the average clr value of all taxa. Taxa were considered keystone when they were ubiquitous and with clr and eigenvector centrality values above these thresholds. Yellow dots represent predicted keystone taxa. **(E)** Venn diagram displaying the comparison of keystone taxa identified in the different networks.

The CAN analysis revealed 60 core associated taxa between OeMG and OeSG networks (Supplementary Figure S1A), and 14 core associated taxa between OmMG and OmSG networks (Supplementary Fig. S1B). The core associations between the SG of the two species consisted of 36 nodes (Supplementary Figure S1C), while core associations of 16 nodes were found between the MG networks of the two species (Supplementary Figure S1D). Seven and 11 taxa were classified as keystone in the MG and SG microbiome of *O. erraticus*, respectively. On the other hand, 16 and seven taxa met the keystone criteria in the microbiome of OmMG and OmSG, respectively (Figures 4A–D; Supplementary Table S4). Two taxa (Muribaculaceae and *Alistipes*) were found as keystone in the microbiome of OeMG, OeSG and OmSG. Fourteen keystone taxa (i.e., *Romboustia*, *Alistipes*, *Ralstonia*, Lachnospiraceae, Lachnospiraceae_NK4A136_group, *Akkermansia*, *Clostridia*_UCG-014, Muribaculaceae, *Lactobacillus*, *Cupriavidus*, *Eubacterium coprostanoligenes* group, *Parabacteroides*, *Dubosiella* and *Bifidobacterium*) were found as nodes in the core association networks.

Local centrality measures were compared between networks using the Jaccard index comparison test. The Jaccard index values of the comparisons OmMG vs. OmSG and OeMG vs. OeSG were lower [P (≤Jacc) < 0.05] and higher [P (≥Jacc) < 0.05] than expected by random, respectively, for all of centrality measures except for betweenness centrality in the OeMG-OeSG comparison which has a random distribution (Table 2). The centrality measures in the MG of the two tick species were lower than expected by random [P (≤Jacc) < 0.05, Table 3], while in the SG they were higher than expected by random [P (≥Jacc) < 0.05, Table 3], except for betweenness centrality, which followed a random distribution (Table 3). The comparison of node clustering between the networks showed the higher Adjusted Rand Index (ARI) value for OeMG compared to OeSG (ARI = 0.60), while the other comparisons showed low similarities in clustering (ARI ≤ 0.40; Table 4).

**Table 2:** Comparison of centrality measures within tick species.

Local centrality measures	OmMG vs. OmSG			OeMG vs. OeSG		
	Jacc^†^	P (≤Jacc)	P (≥Jacc)	Jacc	P (≤Jacc)	P (≥Jacc)
Degree	0.222	0***	1.00	0.529	1.00	0***
Betweenness centrality	0.186	0***	1.00	0.380	0.89	0.14
Closeness centrality	0.222	0***	1.00	0.529	1.00	0***
Eigenvector centrality	0.222	0***	1.00	0.529	1.00	0***
Hub taxa	0.222	0***	1.00	0.529	1.00	0***

^†^Jaccard index. ***p < 0.001.

**Table 3:** Comparison of centrality measures between same tissues in different tick species.

Local centrality measures	OeMG vs. OmMG			OeSG vs. OmSG		
	Jacc^†^	P (≤Jacc)	P (≥Jacc)	Jacc	P (≤Jacc)	P (≥Jacc)
Degree	0.207	0.0e + 00***	1.00	0.382	0.97	0.03*
Betweenness centrality	0.199	1.4e – 05***	0.99	0.287	0.09	0.93
Closeness centrality	0.207	0.0e + 00***	1.00	0.382	0.97	0.03*
Eigenvector centrality	0.207	0.0e + 00***	1.00	0.382	0.97	0.03*
Hub taxa	0.207	0.0e + 00***	1.00	0.382	0.97	0.03*

^†^Jaccard index. ***p < 0.001, *p < 0.05.

**Table 4:** Network clustering comparisons.

Network comparisons	Adjusted rand index (ARI)	*p*-Value
OeMG vs. OeSG	0.60	0
OmMG vs. OmSG	0.14	0
OeMG vs. OmMG	0.18	0
OeSG vs. OmSG	0.40	0

**Figure 5:**
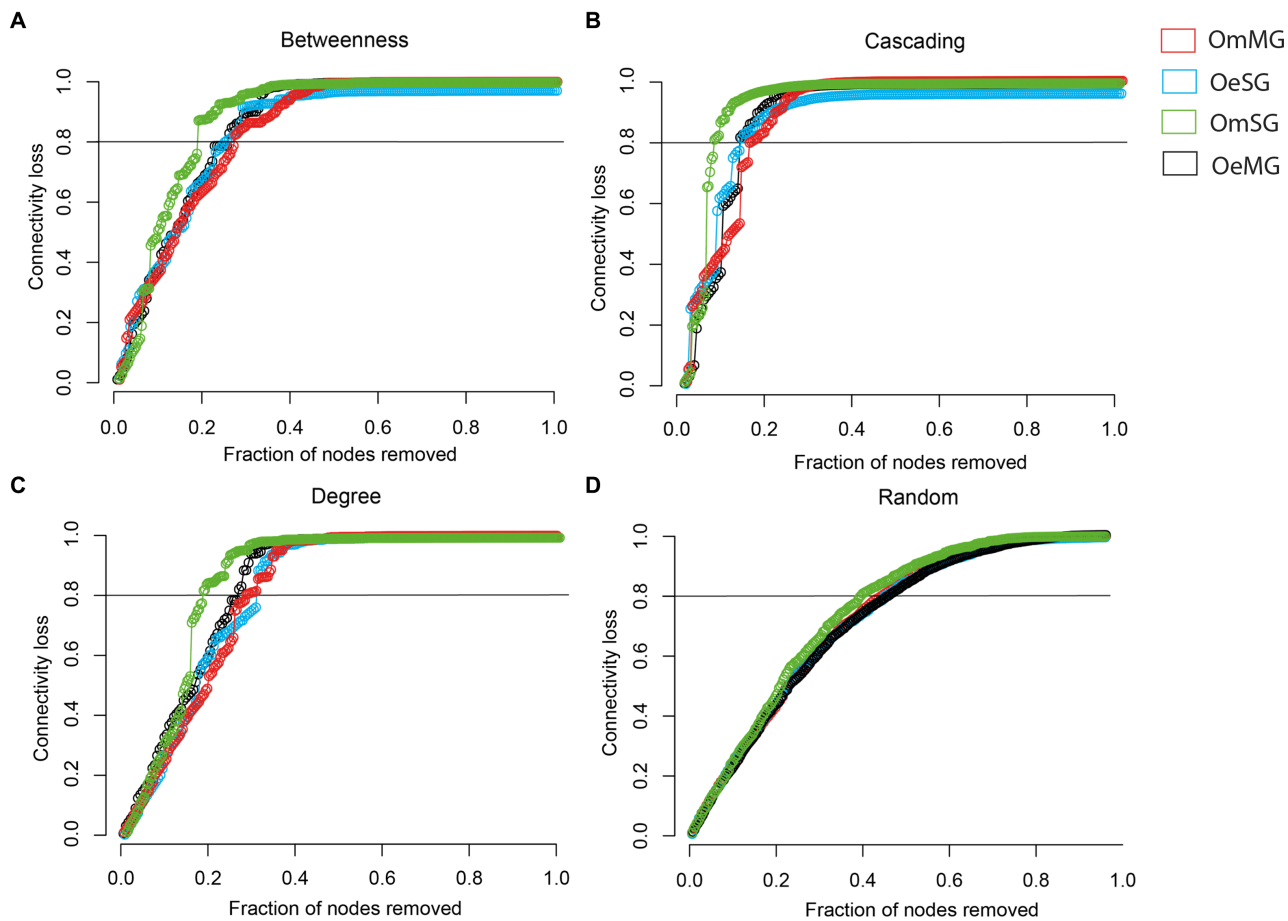
Robustness comparison of OeMG, OeSG, OmMG and OmSG networks. **(A)** Connectivity loss measured after directed attack, removing first the nodes with the highest betweenness centrality. **(B)** Connectivity loss measured after cascading effect, removing nodes with the highest betweenness centrality and recalculating the betweenness centrality value after each removal. **(C)** Connectivity loss measured after intentional attacks that target nodes with very high degree. **(D)** Connectivity loss measured after random removal of taxa in networks.

The robustness of the networks in the tissues of *O. erraticus* and *O. moubata* was analyzed. In all networks, there is a rapid loss of connectivity after a cascading attack, whereas after a random attack, a larger proportion of nodes had to be removed to achieve a complete loss of connectivity (Supplementary Figure S2). After directed attacks by betweenness (Figure 5A), cascading (Figure 5B), and degree (Figure 5C) the OmSG network was less robust than the other networks, as a smaller proportion of nodes removal resulted in a greater loss of connectivity. The OmMG network was more robust after directed attacks by betweenness (Figure 5A) and cascading (Figure 5B), while the OeSG network was more robust to degree attack (Figure 5C). All networks showed a loss of connectivity after a random attack in which a similar proportion of nodes were removed (Figure 5D).

### 3.3. Functional profiles of microbiome in salivary glands and midguts of *Ornithodoros erraticus* and *Ornithodoros moubata*

**Figure 6:**
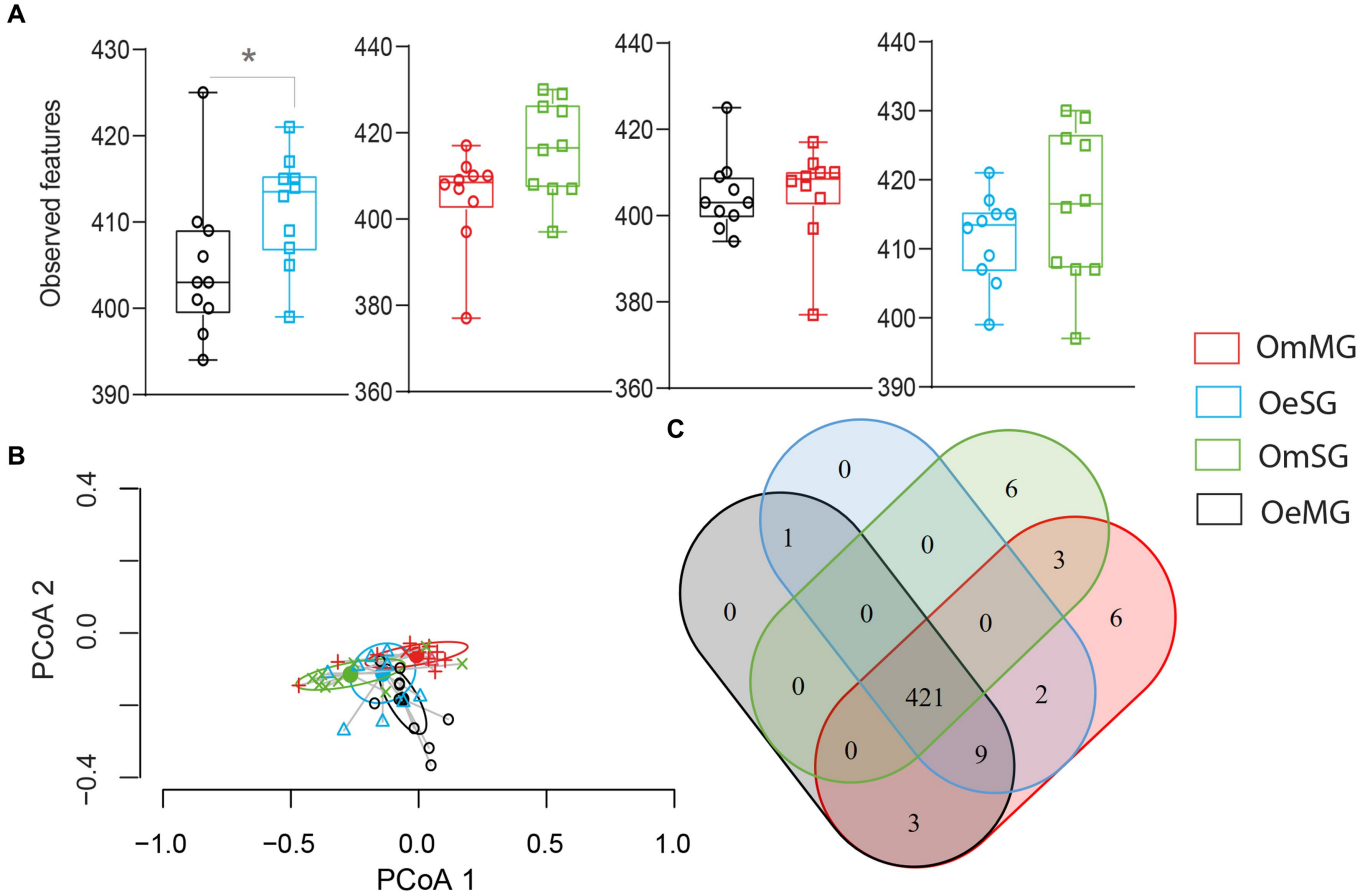
Pathway diversity and composition in the different groups for OeSG, OeMG, OmMG and OmSG samples. **(A)** Observed features **(B)** Comparison of beta diversity with Bray–Curtis dissimilarity index. Beta dispersion of the four datasets. ANOVA test was performed and showed that beta dispersion of the four sets of samples is not significantly different (ANOVA, *p* = 0.83). **(C)** Venn diagram displaying the comparison of pathway.

Changes in composition and structure of the microbial community may affect the inferred functional profile of the tick microbiome. We analyzed and compared the composition, diversity and differential abundance of predicted metabolic pathways in SG and MG of *O. erraticus* and *O. moubata*. Significant differences were found in the observed features of metabolic pathways in the microbiome of OeMG and OeSG (Figure 6A; Supplementary Table S5A). The Bray–Curtis index and a PERMANOVA test showed that the beta diversity of metabolic pathways was significantly different between all datasets (PERMANOVA, *p* < 0.05), except in the SG of the two tick species (Figure 6B; Supplementary Table S5B).

The four datasets shared 421 (93.1%, 452 total) metabolic pathways. Six unique metabolic pathways (1.32%, 452 total) were found in each of the OmSG and OmMG microbiomes (Figure 6C; Supplementary Table S6), while no unique pathway was found for OeSG and OmMG. Three others pathways (0.66%, 452 total) were shared between MG and SG; crotonate fermentation, tylosin biosynthesis and dTDP-6-deoxy-deoxy-α-D-allose biosynthesis. The biphenyl degradation pathway was found in the microbiome of *O. erraticus* (SG-MG; Figure 6C; Supplementary Table S6).

**Figure 7:**
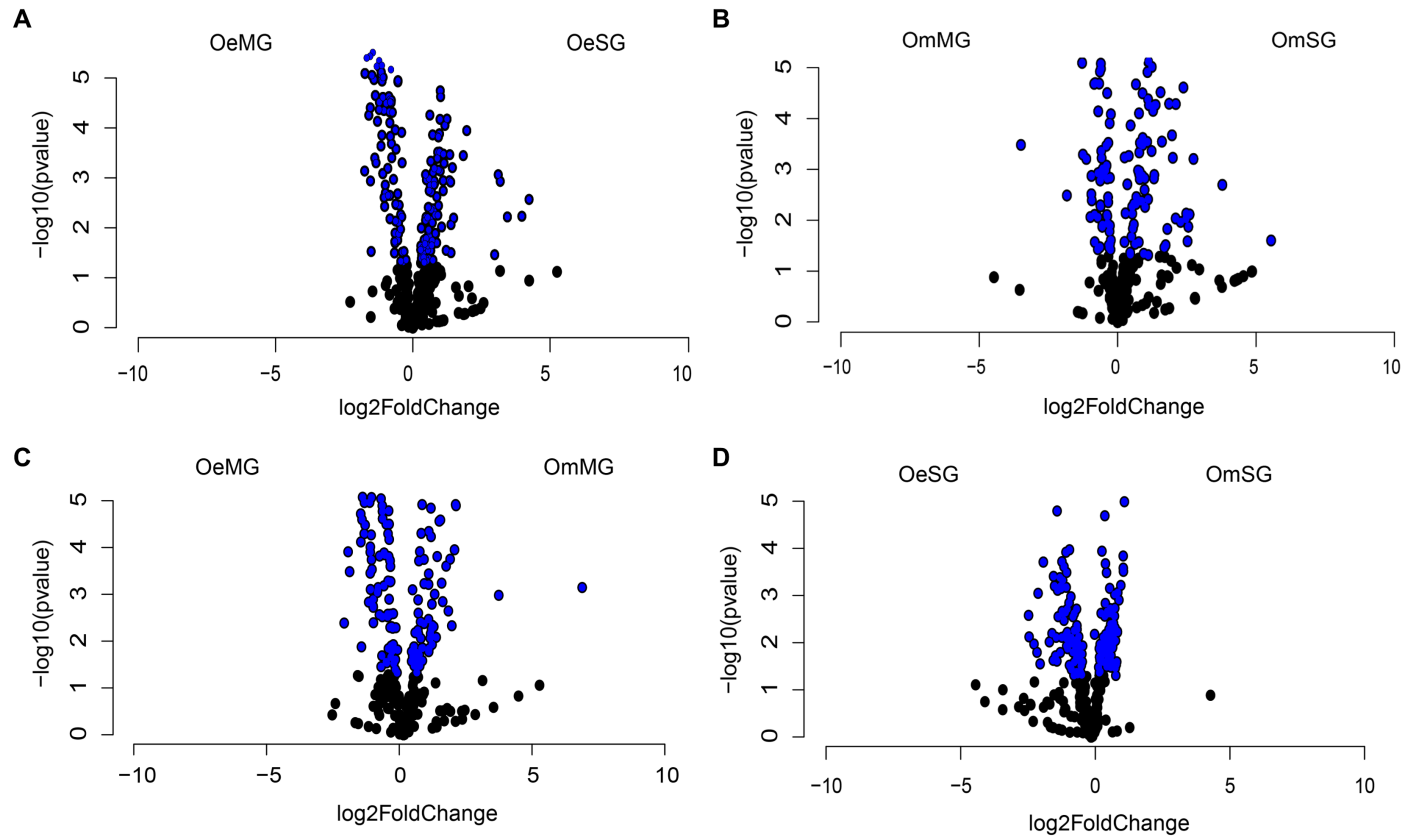
Differential abundance of predicted metabolic pathways. Volcano plot showing the differential abundance of pathways in OeSG-OeMG **(A)**, OmMG-OmSG **(B)**, OeMG-OmMG **(C)** and OmSG-OeSG **(D)**. The pathways with significant differences (Wald test, *p* < 0.05) between the groups are represented by blue dots.

Differential analysis revealed significant changes in the abundance of 146 and 105 metabolic pathways between tissues of *O. erraticus* (Figure 7A) and *O. moubata* (Figure 7B), respectively (Wald test, *p* < 0.05, Supplementary Table S7). In the MG (Figure 7C) and SG (Figure 7D) of the two species, 122 and 189 pathways, respectively, were found with significant changes in abundance (Wald test, *p* < 0.05, Supplementary Table S7).

## 4. Discussion

The composition and variability of the tick microbiome have been associated with several factors including tick species, tissue type, sex, stage and environmental factors, among others (Wu-Chuang et al., 2021). In our study, the use of ticks of the same sex (i.e., female) reared in colonies and fed on the same host (i.e., rabbits) is expected to reduce confounding factors. The reduction of covariables to test our hypothesis (i.e., the microbial communities in *O. erraticus* and *O. moubata* microbiome are tissue-specific) is a strength of our work. However, a limitation of this study is the absence of negative controls that would have allowed us to exclude potential bacterial contaminants from the analysis and obtain a better resolution of the microbial communities in the soft ticks studied. Contamination throughout the different stages of the analytical process, from DNA extraction to amplification, can potentially affect analysis of the DNA sequences as well as the interpretation of the results (Lejal et al., 2020). However, it is noteworthy mentioning that the disinfection process used in our study may have reduced potential tick surface contaminants.

Our analysis on diversity, relative abundance and composition revealed that the bacterial communities in *O. erraticus* and *O. moubata* are species and tissue-specific. These results are consistent with previous microbiome studies on hard and soft ticks. Chicana et al. (2019) found significant differences in bacterial diversity and composition between different hard tick species including *Ixodes pacificus*, *Ixodes angustus*, *Dermacentor variabilis*, *Dermacentor occidentalis*, *Dermacentor albipictus*, and *Haemaphysalis leporispalustris*. Rojas-Jaimes et al. (2022) found differences in bacterial diversity and richness between species of the genus *Amblyomma* collected from different hosts. Particularly, the bacterial microbiome of *Amblyomma scalpturatum* and *Amblyomma ovale* collected from *Tapirus terrestris* and *Amblyomma sabanerae* collected from *Chelonoidis denticulate* presented significant differences in diversity (Rojas-Jaimes et al., 2022). Differences in microbial community diversity, composition and relative abundance were also found in the soft tick species including *Ornithodoros cf. hasei* (Carvajal-Agudelo et al., 2022), *Ornithodoros turicata* (Barraza-Guerrero et al., 2020), *Ornithodoros maritimus* (Gomard et al., 2021) and *Argas japonicus* (Yan et al., 2019). Differences in bacterial diversity, composition and relative abundance were also found in MG and SG of *Dermacentor andersoni* (Gall et al., 2017), *Dermacentor silvarum* (Duan et al., 2020) and *Ixodes ricinus* (Wiesinger et al., 2023). Differences in bacterial diversity and composition between tissues of the same tick species could be associated to differential microbe-microbe interactions (e.g., competition caused by differences in nutrient availability) and/or host–microbe interactions (e.g., tissue-specific immunity or immune evasion by microbes; Wu-Chuang et al., 2021). In our study, several genera of Proteobacteria (i.e., *Rickettsiella*, *Cellvibrio*, *Methylophaga*, Xanthomonadaceae, *Cupriavidus*, and *Ralstonia*) presented significant changes in relative abundance between the four datasets. Kumar et al. (2022) showed a significant difference in the relative abundance of Proteobacteria in SG and MG of unfed and partially engorged *Ixodes scapularis* ticks. In addition, Proteobacteria was also reported as the most abundant taxon in soft and hard ticks such as *A. japonicus* (Yan et al., 2019), *O. cf. hasei* (Carvajal-Agudelo et al., 2022), *Dermacentor marginatus*, *I. ricinus* and *Rhiphicephalus sanguineus* (Portillo et al., 2019). The abundance of Proteobacteria in different hard and soft tick species may be associated to the fact that the bacterial species within the ecosystem use a wide range of metabolic strategies (Zhou et al., 2020), which may decrease competition with other microorganisms for nutrient availability allowing them to survive in diverse environments. Altogether, our study supports high taxonomic variability in hard and soft tick microbiome (Wu-Chuang et al., 2021).

Bacterial assembly within arthropod vectors influence pathogen ecology and transmission (Aželytė et al., 2022). The detection of bacterial taxa in the same physical space (i.e., tick tissues) could be an indication of direct interactions between microbes (Gomard et al., 2021). Here, bacterial co-occurrence networks, a methodological framework to construct ideally defined associations, were used to characterize the bacterial associations in SG and MG of *O. erraticus* and *O. moubata*. Network analysis showed that positive correlations were more frequent in all microbiomes except for OeMG where negative correlations were more frequent. The unequal number of positive and negative interactions between *O. erraticus* and *O. moubata* suggests differences in microbial-microbial interactions in these two species. Positive associations could indicate cooperation and/or functional complementarity between different bacterial taxa in a shared niche within the host organism, while negative associations would indicate co-exclusion or competition between bacterial taxa (Kumar et al., 2022). Our results suggest that microbial competition in the MG of soft ticks is stronger in *O. erraticus* compared to *O. moubata*. Differences in community assembly in the MG of *O. moubata* and *O. erraticus* concur with dissimilarities in the MG transcriptome (Oleaga et al., 2017a, 2018) and the anatomo-physiology of the MG in these two species. Particularly, the digestive tube of *O. moubata* lacks anatomical connection between the MG and the hindgut, which prevents the passage of haematin and other toxic digestion waste to the hindgut to be excreted (Sonenshine and Roe, 2014). Different concentrations of haematin and other digestion waste may explain differences in the bacterial community assembly in the MG of *O. erraticus* and *O. moubata*. To further characterize conserved patterns of microbial interactions, a CAN analysis was performed. Results showed the existence of core associations in networks of different species and tissues. Despite differences in the networks of OeMG and OmMG, we found the existence of a conserved CAN in the MG of *O. erraticus* and *O. moubata*, suggesting conserved patterns of microbe-microbe interactions in the microbiome of the genus *Ornithodoros*. It is important to mention that since microbial networks alone do not provide information on the biological nature of the microbial interactions; experimental studies are needed to specifically test the biological basis of the positive and negative interactions between the taxa encountered.

Networks can also be compared in terms of their resistance to taxa removal. In our study, we used robustness, an important property of networks (Liu et al., 2017), to test the ability of the networks to withstand directed and random attacks. Loss in connectivity after directed attacks was comparable between all networks; however OmSG network displayed the lowest robustness. Significant reduction of network robustness after directed attacks was found in the microbiota of mice exposed to antibiotics (Mahana et al., 2016). This analysis suggests that specific factors decreasing network robustness might be readout of ecosystem collapse (Mahana et al., 2016). The lower robustness suggests higher susceptibility of OmSG microbiome to disturbing factors. A previous report showed that network robustness decreased only marginally in *I. scapularis* ticks infected with the tick-borne pathogen *Anaplasma phagocytophilum* (Estrada-Peña et al., 2020a), compared with other disturbance factors such as anti-tick immunity (Estrada-Peña et al., 2020a), or anti-microbiota vaccines (Mateos-Hernández et al., 2021), which perturbed the network significantly. Altogether, the results suggest that OmSG microbiome might be highly susceptible to perturbation by anti-microbiota vaccines targeting keystone taxa (see below). In addition to co-occurrence patterns, microbial networks can be used to identify keystone taxa (Banerjee et al., 2018). Keystone species play an important role in the stability of microbial ecosystems. In our study, keystone taxa were identified based on their ubiquitousness, high relative abundance, and high eigenvector centrality in the networks, as previously reported for ticks (Mateos-Hernández et al., 2020; Maitre et al., 2022). Most keystone taxa identified in the microbiome of *O. erraticus* and *O. moubata* were tissue-specific, while others were found in both the MG and SG of each species (Supplementary Table 4). Identification of the same keystone taxa in both tissues suggests the possibility of migration of key microbes between these different niches. Notably, Muribaculaceae and *Alistipes* were found as keystone taxa in the microbiome of OeMG, OeSG and OmSG. Four keystone taxa were identified in *I. scapularis* ticks exposed to different temperatures (Wu-Chuang et al., 2022b), and the functional diversity of predicted metabolic pathways of the tick microbiome was contained in these keystone bacteria and their associated taxa (Wu-Chuang et al., 2022b). The involvement of the keystone Muribaculaceae and *Alistipes* and their associated taxa in the functional stability of the microbial communities in OeMG, OeSG and OmSG guarantees further research. In addition, these two taxa could be used as candidates in anti-microbiota vaccine formulations. Previous studies showed that targeting *I. ricinus* microbiome caused significant mortality in ticks fed on α-1,3-galactosyltransferase-deficient-C57BL/6 mice immunized against the keystone taxon *Escherichia*-*Shigella* (Mateos-Hernández et al., 2020). Furthermore, immunization against *Escherichia*-*Shigella* elicited the production of host antibodies the modulated the tick microbiome (Mateos-Hernández et al., 2021).

The metabolic activity of bacterial populations in specific tissues could influence tick physiology. Differences in the beta diversity and abundance of predicted metabolic pathways suggest functional specialization between SG and MG within the same species. Differential gene expression (Oleaga et al., 2017a, 2018) and protein representation (Oleaga et al., 2017b, 2021; Pérez-Sánchez et al., 2022) between MG (Oleaga et al., 2017a,b, 2018) and SG (Oleaga et al., 2021; Pérez-Sánchez et al., 2022) of *O. erraticus* (Oleaga et al., 2018; Pérez-Sánchez et al., 2022) and *O. moubata* (Oleaga et al., 2017a,b, 2021) demonstrates functional differentiation and specialization in these tissues, which may be influenced by the composition of the microbial community and its functional contribution as a result of its metabolic activity. Despite evidence of metabolic specialization in the SG and MG of *O. erraticus* and *O. moubata*, 93.1% of the predicted metabolic pathways were shared by all the datasets. Likewise, a previous study found similar biochemical pathway composition across tick ontogeny (Zolnik et al., 2016), suggesting high functional redundancy in the tick microbiome (Estrada-Peña et al., 2020a,b).

## 5. Conclusion

This study shows that the structure and composition of the tick microbiome in the soft ticks *O. erraticus* and *O. moubata* is species and tissue specific. Ticks may be exposed to different bacterial species; but, this does not guarantee colonization. Biological interactions exist between the bacteria that are part of the tick microbiome. Aspects such as the availability of nutrients or the ability to evade the immune response in the different tissues of the tick, may explain the differences in diversity and abundance of the bacterial community. Using bacterial assemblies, we were able to characterize the bacterial associations in SG and MG of *O. erraticus* and *O. moubata*, and these were found to depend on tick species and tissue. Bacterial taxa were detected in the same tissue, which could indicate a direct association from a biological point of view. The robustness of the OmMG and OeSG networks suggests that there is no trophic dependence or competition between coexisting genera, while the similarity of biochemical pathways suggests that the loss of bacterial taxa does not result in significant changes in microbial function. Pathogens and other microorganisms coexist within ticks, and bacterial communities residing inside ticks can modulate their vectorial capacity by affecting tissue colonization by pathogens (Wu-Chuang et al., 2021). Studies on the composition of internal microbial communities provide the basis for developing new strategies to interrupt pathogen transmission by modulating the tick microbiome. For example, keystone taxa identified in the microbiome of *I. ricinus* were used as vaccine candidates to reduce tick infestation (Mateos-Hernández et al., 2020) and modulate the tick microbiome (Mateos-Hernández et al., 2021). The finding of Muribaculaceae and *Alistipes* as keystone taxa in the SG of the two tick species and the MG of *O. erraticus* justify their use as anti-microbiota vaccine candidates to target all tissues and/or for the tissue-specific manipulation to block pathogen transmission. Analysis of the differences in bacterial community composition in different tick tissues could be useful to determine the major key players in pathogen colonization and to make the manipulation of the tick microbiome more specific.

## Data availability statement

The datasets presented in this study can be found in online repositories. The names of the repository/repositories and accession number(s) can be found at: https://www.ncbi.nlm.nih.gov/, PRJNA931807.

## Author contributions

EP-S: formal analysis, visualization and writing-original draft. AC-A: investigation, review and editing. AM: formal analysis and review and editing. AW-C: formal analysis and writing-review and editing. LM-H: data curation, writing-review and editing. AC: writing-review and editing. DO: software, writing-review and editing. AO: investigation and writing-review and editing. RP-S: conceptualization, resources, supervision and writing-original draft. AC-C: conceptualization, resources, visualization, supervision and writing-original draft. All authors contributed to the article and approved the submitted version.

## Funding

UMR BIPAR was supported by the French Government’s Investissement d’Avenir program, Laboratoire d’Excellence “Integrative Biology of Emerging Infectious Diseases” (grant no. ANR-10-LABX-62-IBEID). AW-C was supported by Programa Nacional de Becas de Postgrado en el Exterior “Don Carlos Antonio López” (grant no. 205/2018). AM was supported by the ‘Collectivité de Corse’, grant: ‘Formations superieures’ (grant code. SGCE-RAPPORT No. 0300). AC-A was supported by project “CLU-2019-05-IRNASA/CSIC Unit of Excellence,” granted by the Junta de Castilla y León and co-financed by the European Union (ERDF “Europe drives our growth”).

## Conflict of interest

The authors declare that the research was conducted in the absence of any commercial or financial relationships that could be construed as a potential conflict of interest.

## Publisher’s note

All claims expressed in this article are solely those of the authors and do not necessarily represent those of their affiliated organizations, or those of the publisher, the editors and the reviewers. Any product that may be evaluated in this article, or claim that may be made by its manufacturer, is not guaranteed or endorsed by the publisher.
