# Supplementary figures and images for: Comparison of salivary gland and midgut microbiome in the soft ticks Ornithodoros erraticus and Ornithodoros moubata
Source: Front Microbiol. 2023 May 9;14:1173609. doi: 10.3389/fmicb.2023.1173609 (PMC10203192; doi:10.3389/fmicb.2023.1173609)

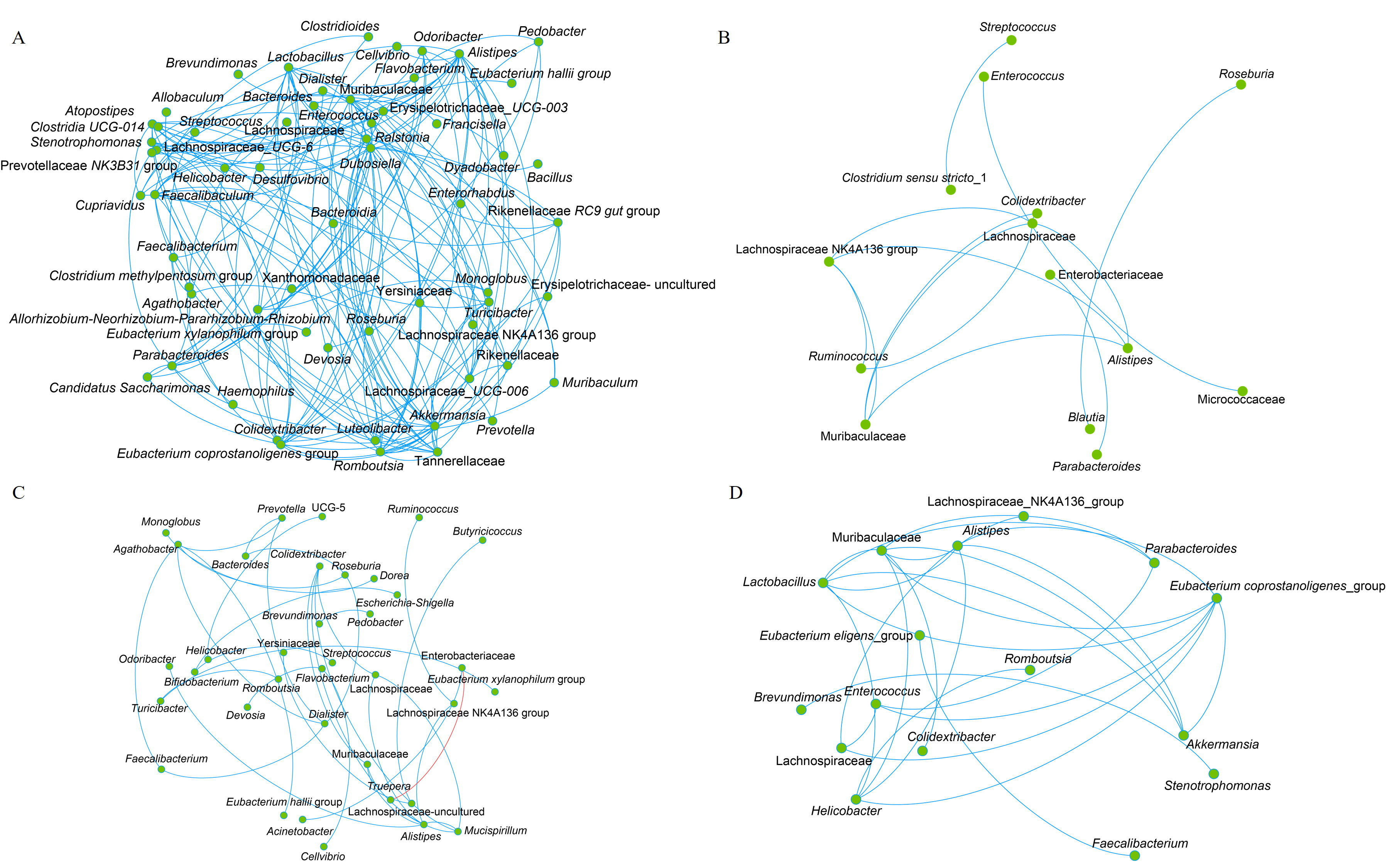

Supplement: Supplementary file 1 [file Image_1.PNG]

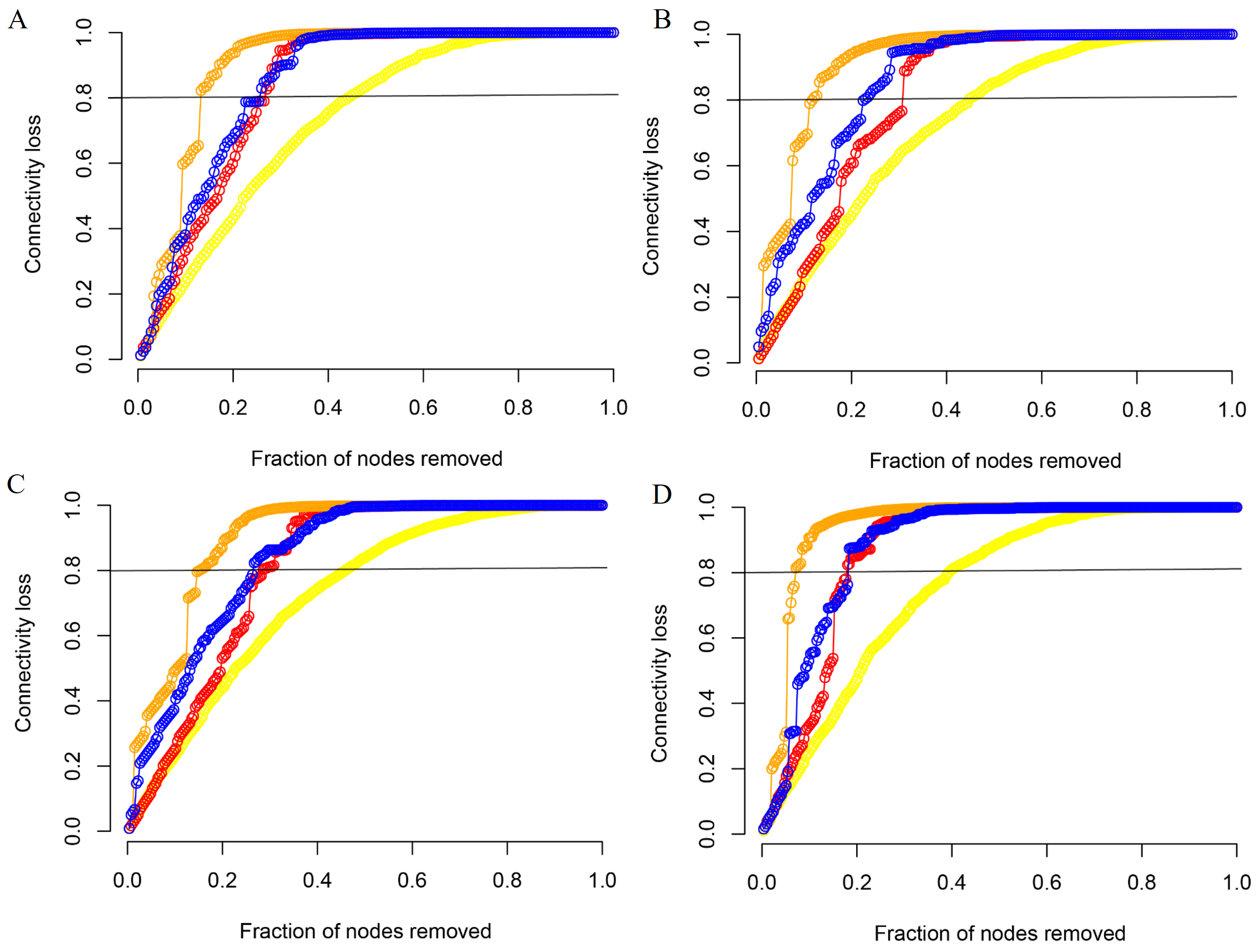

Supplement: Supplementary file 2 [file Image_2.TIF]
